# Stem Cells as *In Vitro* Models of Disease

**DOI:** 10.1155/2012/565083

**Published:** 2012-12-30

**Authors:** Mirella Dottori, Mary Familari, Stefan Hansson, Kouichi Hasegawa

**Affiliations:** ^1^The University of Melbourne, Parkville, VIC 3010, Australia; ^2^Lund University, 221 85 Lund, Sweden; ^3^Institute for Integrated Cell-Material Science, Kyoto University, Kyoto 606-8501, Japan

At the present time, for many human diseases, medical research predominantly relies on having appropriate model systems to study disease states and in order to develop therapies. Nonhuman animal models, particularly transgenics, have allowed us to recreate disease states *in vivo*. However, there is still a need to have corresponding *in vitro* model systems to study pathogenesis at the cellular level as well as for fast-tracking discoveries of therapeutic compounds. Ideally, an *in vitro* disease model would be established from human diseased tissue that can demonstrate relevant degenerative mechanisms. The tremendous advances within the field of stem cell biology over the last decade may now make *in vitro* models possible for certain diseases that were previously thought unlikely.

This special issue in *Stem Cells International* is a collection of review articles that describe various ways in which stem cells have be utilized to create *in vitro* disease models.

The Noble-prize winning discoveries in reprogramming have given scientists the opportunity to generate stem cells from any disease type. Whilst this technology has opened new doors for creating *in vitro* model systems, challenges still remain including how to differentiate pluripotent stem cells to generate stage-specific (whether progenitor or mature stage) and homogeneous cell types that are relevant to the disease. A significant aspect of that relies on the development of *in vitro* assays to study the cellular function. N. Kawaguchi et al. highlight these issues with respect to using pluripotent stem cells to model cardiac disease and channelopathies.

Reprogramming technology is highly useful for creating *in vitro* disease models that are caused by known genetic mutations. The review by L. Linta et al. describes how neurons derived from pluripotent stem cells carrying mutations in alpha-synuclein or *LRRK2* genes show pathological characteristics of synucleinopathies. However, for many diseases, the causes are idiopathic and interrogation of specific cell types in isolation may not elucidate other potential environmental triggers. When there are multifactorial causes, such as those for Parkinson's disease as reviewed by P. L. Martínez-Morales and I. Liste, then it is advantageous to establish multiple stem cell lines from different patients, including both idiopathic and specific genetic mutant conditions.

Parallel to the development of reprogramming technologies, the field of stem cell biology has also rapidly expanded through the identification of stem cell niches within most adult tissues. Isolation, maintenance, and expansion of stem cell/progenitor pools within a tissue provide an alterative source of cells that can be used to model disease conditions, particularly of that tissue. The review by R. J. Medina et al. describes how a subpopulation of endothelial progenitor cells isolated from human blood can be used to model vascular disease.

For some diseases, especially certain cancers, pathological mechanisms may begin within the stem cell population, perhaps due to DNA damage. In these scenarios, isolation of tissue-specific stem cells is used to model disease genesis. The review by A. Gutiérrez-Rivera et al. explains how skin-derived precursor cells may be the cells of origin in neurofibromatosis type 1 tumours. J. D. Hoerter et al. also provide a thorough review on how melanomas may arise from damaged extrafollicular melanocyte stem cells.

In summary, this special issue provides a broad spectrum of how stem cells can offer innovative and novel approaches to study disease mechanisms. We would like to thank all of the authors, reviewers, Guest Editors, and Editor-in-Chief for their great contributions and support towards this special issue.

*Mirella Dottori* *Mirella Dottori* *Mary Familari* *Mary Familari* *Stefan Hansson* *Stefan Hansson* *Kouichi Hasegawa* *Kouichi Hasegawa*

